# Supplementary material for: Effects of Hydrothermal Processing on Volatile and Fatty Acids Profile of Cowpeas (Vigna unguiculata), Chickpeas (Cicer arietinum) and Kidney Beans (Phaseolus vulgaris)
Source: Molecules. 2022 Nov 24;27(23):8204. doi: 10.3390/molecules27238204 (PMC9739933; doi:10.3390/molecules27238204)
Supplement: Supplementary file 1 [file molecules-27-08204-s001.zip › molecules-2002659-supplementary.pdf]

Supplementary Materials

Effects of Hydrothermal Processing on Fatty Acids and Volatile Profile of Cowpeas (*Vigna unguiculata*), Chickpeas (*Cicer arietinum*) and Kidney Beans (*Phaseolus vulgaris*): A Fingerprinting Approach

Prit Khrisanapant [1,2], Biniam Kebede [1,*], Sze Ying Leong [1,2] and Indrawati Oey [1,2,*]

[1] Department of Food Science, University of Otago, Dunedin 9054, New Zealand
[2] Riddet Institute, Palmerston North 4442, New Zealand
* Correspondence: biniam.kebede@otago.ac.nz (B.K.); indrawati.oey@otago.ac.nz (I.O.); Tel.: +64-3-479-7257 (B.K)

Table S1. Commercial standards for fatty acid methyl ester analysis

Analyte	CAS Number	Target Concentration
Butyric acid methyl ester	623-42-7	400 µg/mL
Caproic acid methyl ester	106-70-7	400 µg/mL
Caprylic acid methyl ester	111-11-5	400 µg/mL
Capric acid methyl ester	110-42-9	400 µg/mL
Undecanoic acid methyl ester	1731-86-8	200 µg/mL
Lauric acid methyl ester	111-82-0	400 µg/mL
Tridecanoic acid methyl ester	1731-88-0	200 µg/mL
Myristic acid methyl ester	124-10-7	400 µg/mL
Myristoleic acid methyl ester	56219-06-8	200 µg/mL
Pentadecanoic acid methyl ester	7132-64-1	200 µg/mL
cis-10-Pentadecenoic acid methyl ester	90176-52-6	200 µg/mL
Palmitic acid methyl ester	112-39-0	600 µg/mL
Palmitoleic acid methyl ester	1120-25-8	200 µg/mL
Heptadecanoic acid methyl ester	1731-92-6	200 µg/mL
cis-10-Heptadecenoic acid methyl ester	75190-82-8	200 µg/mL
Stearic acid methyl ester	112-61-8	400 µg/mL
Elaidic acid methyl ester	1937-62-8	200 µg/mL
Oleic acid methyl ester	112-62-9	400 µg/mL
Linolelaidic acid methyl ester	2566-97-4	200 µg/mL
Linoleic acid methyl ester	112-63-0	200 µg/mL
Arachidic acid methyl ester	1120-28-1	400 µg/mL
g-Linolenic acid methyl ester	16326-32-2	200 µg/mL
cis-11-Eicosenoic acid methyl ester	2/09/2390	200 µg/mL
Linolenic acid methyl ester	301-00-8	200 µg/mL
Heneicosanoic acid methyl ester	6064-90-0	200 µg/mL
cis-11,14-Eicosadienoic acid methyl ester	7/02/2463	200 µg/mL
Behenic acid methyl ester	929-77-1	400 µg/mL
Methyl cis-8,11,14-eicosatrienoate	21061-10-9	200 µg/mL
Erucic acid methyl ester	1120-34-9	200 µg/mL
cis-11-14-17-Eicosatrienoic acid methyl ester	55682-88-7	200 µg/mL
Arachidonic acid methyl ester	2566-89-4	200 µg/mL
Tricosanoic acid methyl ester	2433-97-8	200 µg/mL

Analyte	CAS Number	Target Concentration
cis-13,16-Docosadienoic acid methyl ester	61012-47-3	200 µg/mL
Lignoceric acid methyl ester	2442-49-1	400 µg/mL
cis-5,8,11,14,17-Eicosapentaenoic acid methyl ester	2734-47-6	200 µg/mL
Nervonic acid methyl ester	2733-88-2	200 µg/mL
cis-4,7,10,13,16,19-Docosahexaenoic acid methyl ester	301-01-9	200 µg/mL

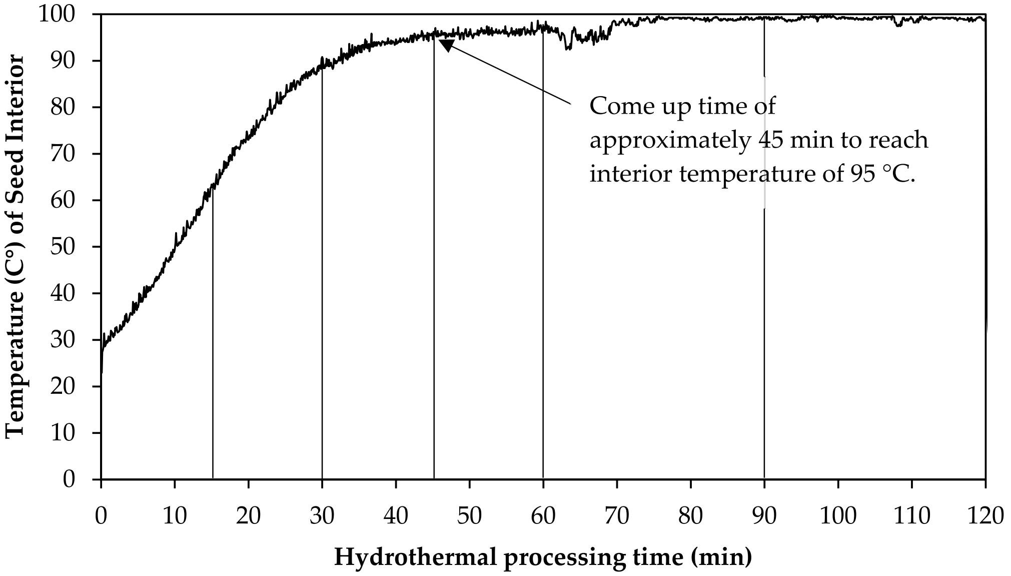

Figure S1. Time-temperature profile of seed interior during hydrothermal processing.

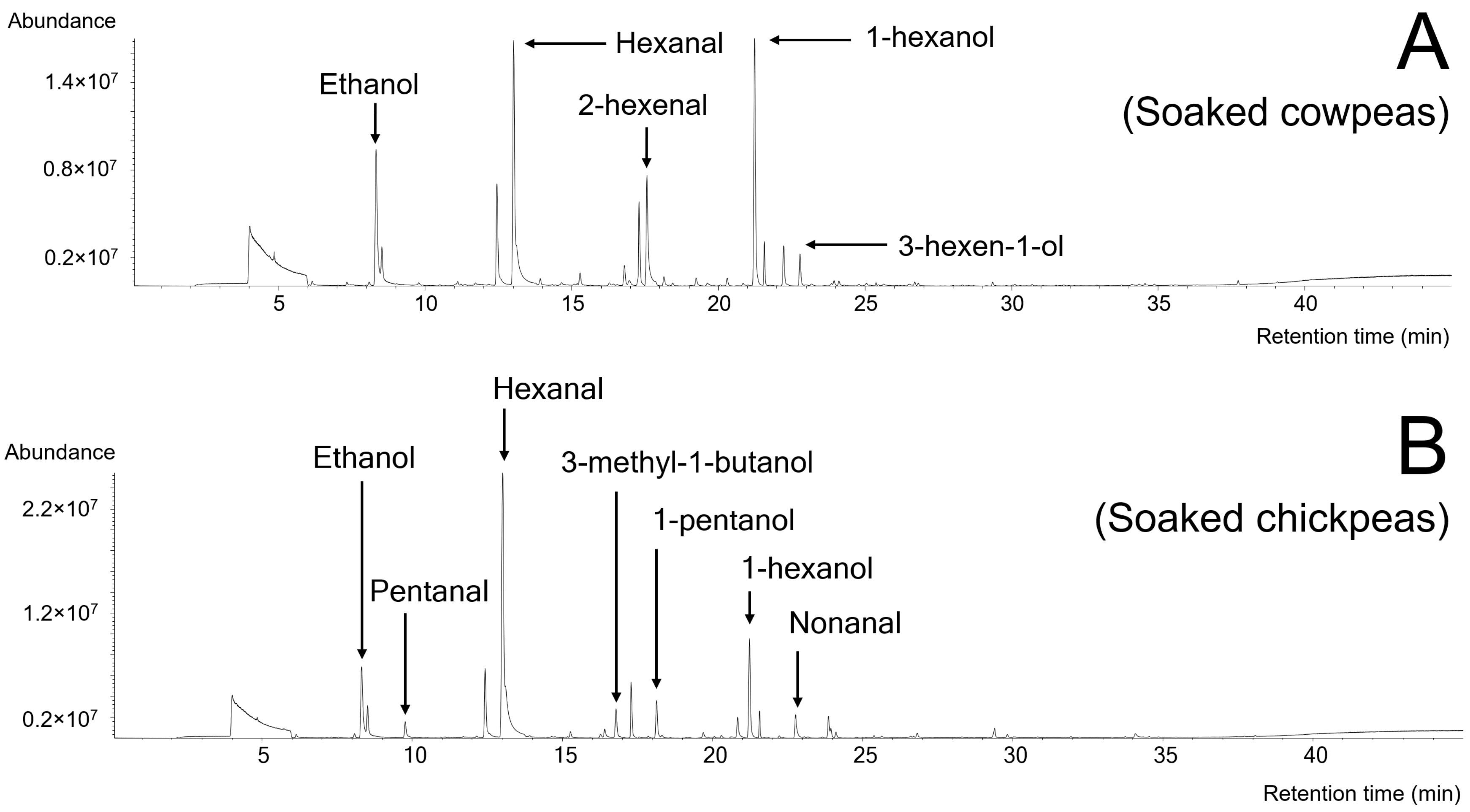

Abundance
1.4×10^7
0.8×10^7
0.2×10^7
Ethanol
Hexanal
2-hexenal
1-hexanol
3-hexen-1-ol
A
(Soaked cowpeas)
5
10
15
20
25
30
35
40
Retention time (min)
Abundance
2.2×10^7
1.2×10^7
0.2×10^7
Ethanol
Pentanal
Hexanal
3-methyl-1-butanol
1-pentanol
1-hexanol
Nonanal
B
(Soaked chickpeas)
5
10
15
20
25
30
35
40
Retention time (min)

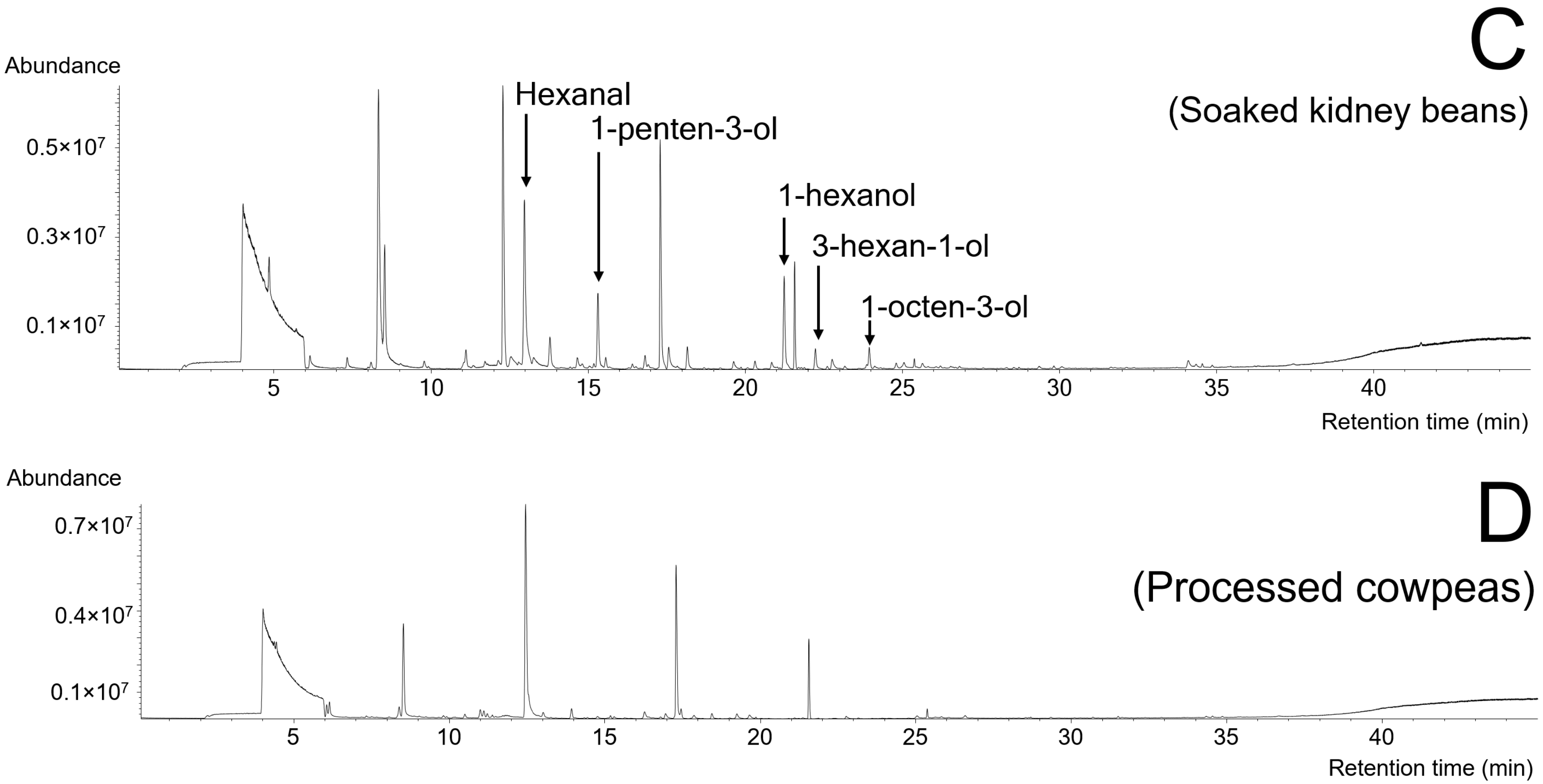
C
(Soaked kidney beans)
Abundance
0.5×10^7
0.3×10^7
0.1×10^7
Hexanal
1-penten-3-ol
1-hexanol
3-hexan-1-ol
1-octen-3-ol
5
10
15
20
25
30
35
40
Retention time (min)
D
(Processed cowpeas)
Abundance
0.7×10^7
0.4×10^7
0.1×10^7
5
10
15
20
25
30
35
40
Retention time (min)

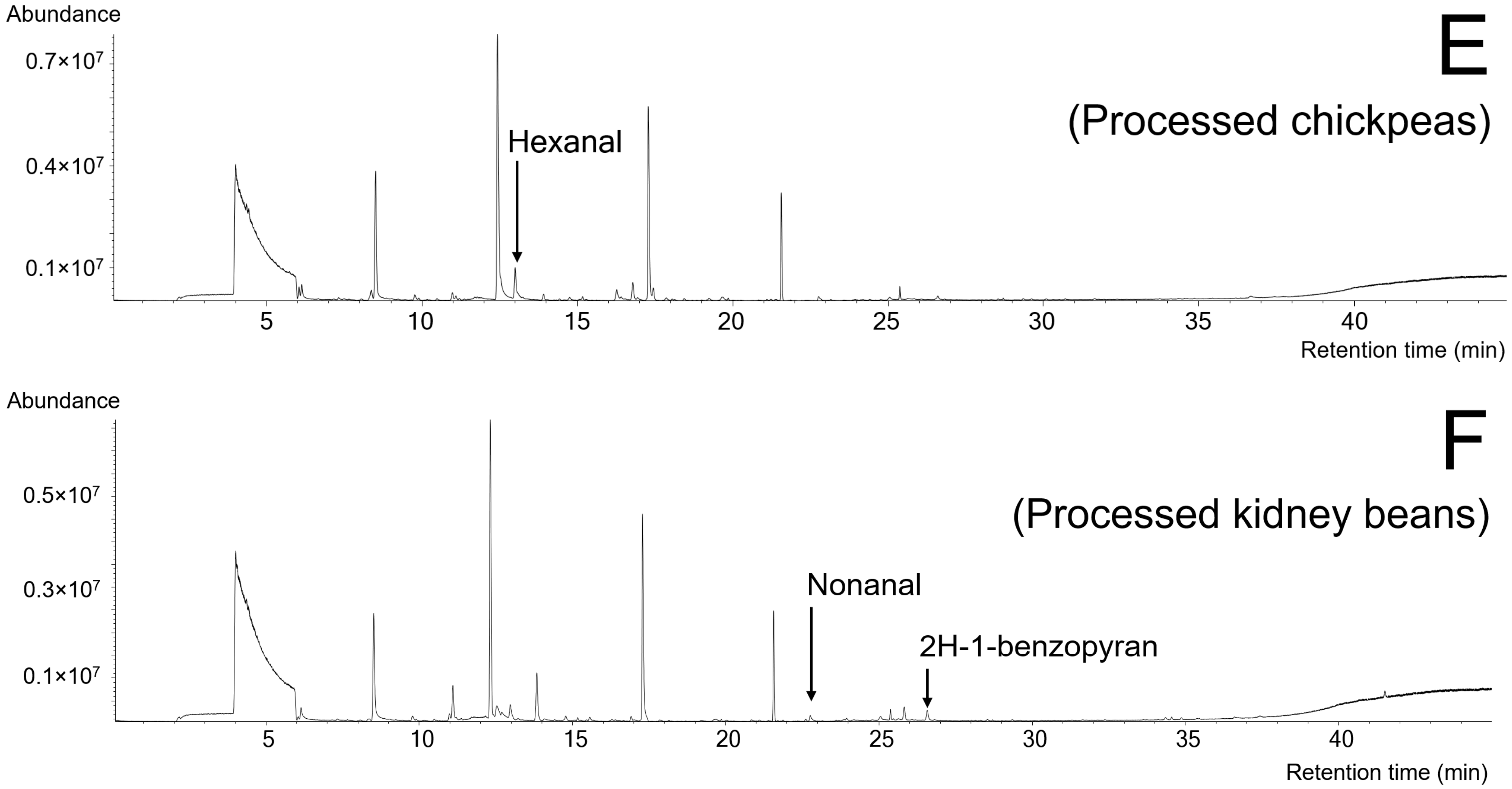

Figure S2. *Representative total ion chromatograms of cowpeas, chickpeas and kidney beans in the opened system approach. A=soaked cowpeas; B=soaked chickpeas; C=soaked kidney beans; D=processed cowpeas; E=processed chickpeas; F=processed kidney beans.*

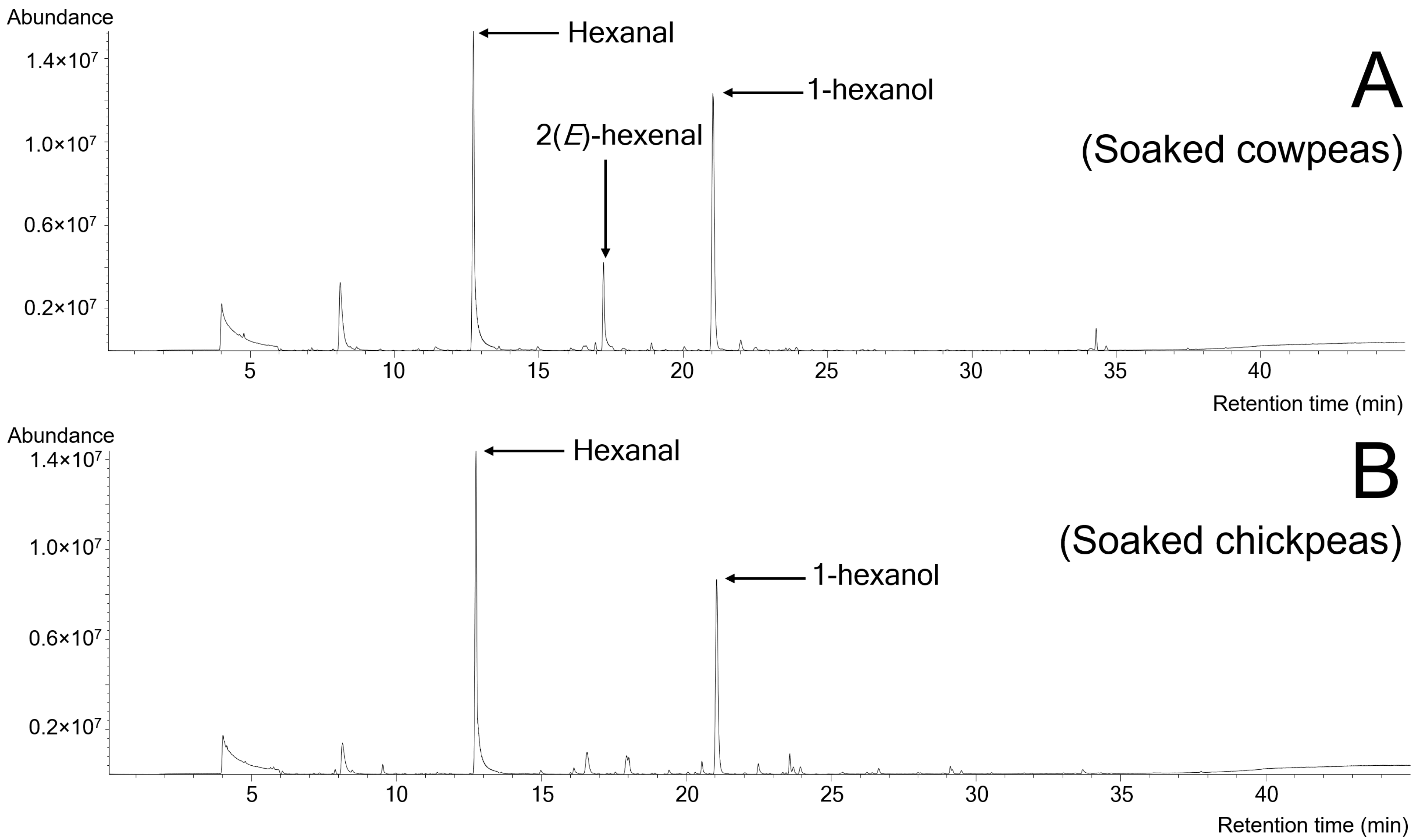

Abundance
1.4×10^7
1.0×10^7
0.6×10^7
0.2×10^7
Hexanal
2(E)-hexenal
1-hexanol
A
(Soaked cowpeas)
5
10
15
20
25
30
35
40
Retention time (min)
Abundance
1.4×10^7
1.0×10^7
0.6×10^7
0.2×10^7
Hexanal
1-hexanol
B
(Soaked chickpeas)
5
10
15
20
25
30
35
40
Retention time (min)

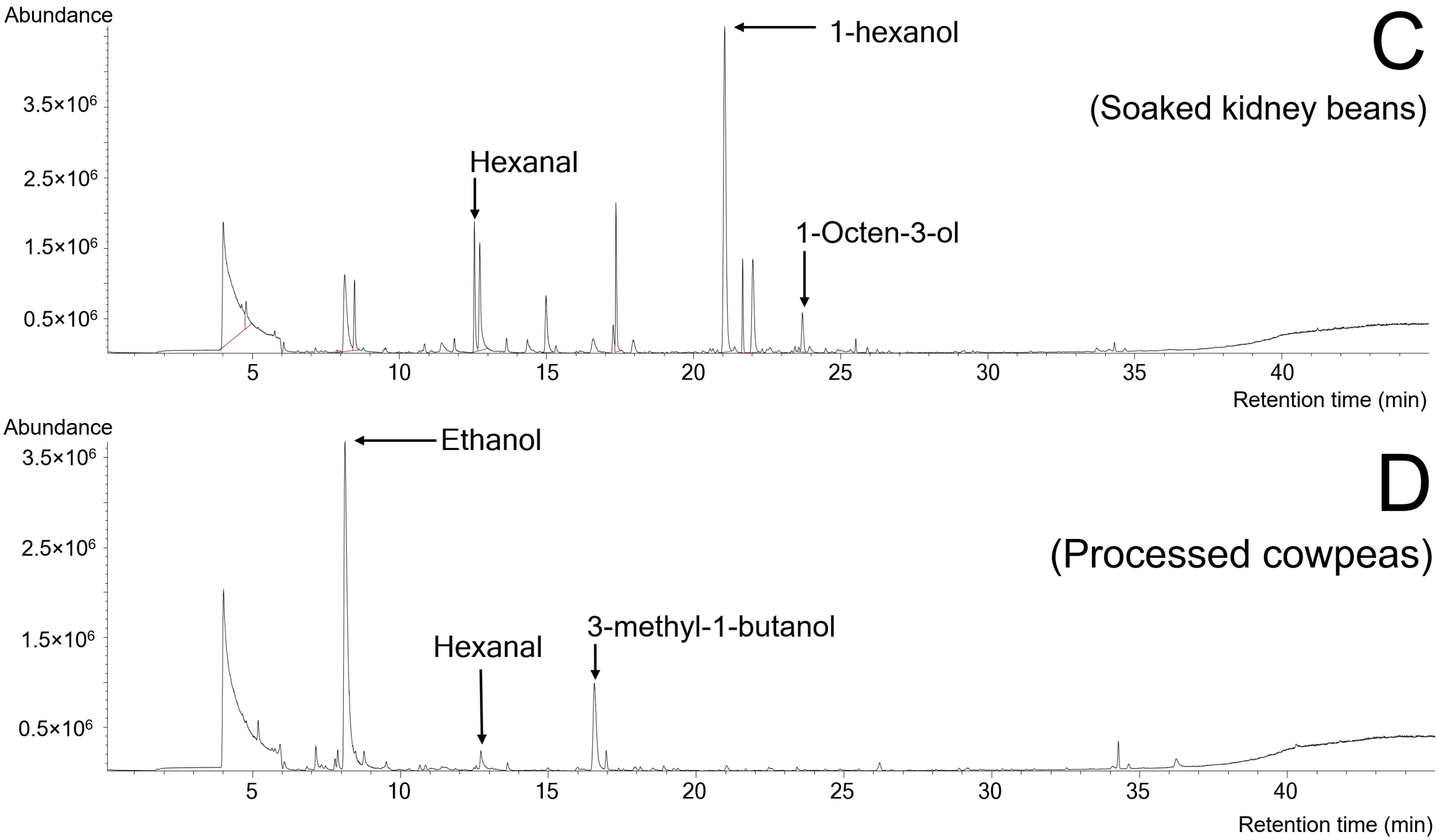

Abundance
3.5×10^6
2.5×10^6
1.5×10^6
0.5×10^6
1-hexanol
C
(Soaked kidney beans)
Hexanal
1-Octen-3-ol
5
10
15
20
25
30
35
40
Retention time (min)
Abundance
3.5×10^6
2.5×10^6
1.5×10^6
0.5×10^6
Ethanol
D
(Processed cowpeas)
Hexanal
3-methyl-1-butanol
5
10
15
20
25
30
35
40
Retention time (min)

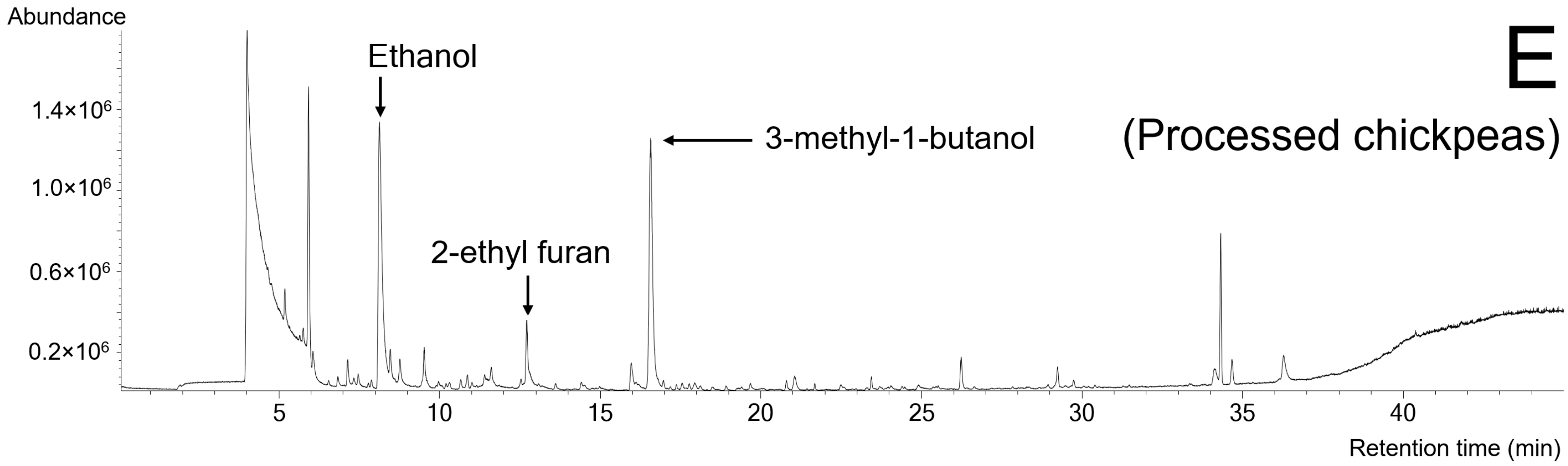

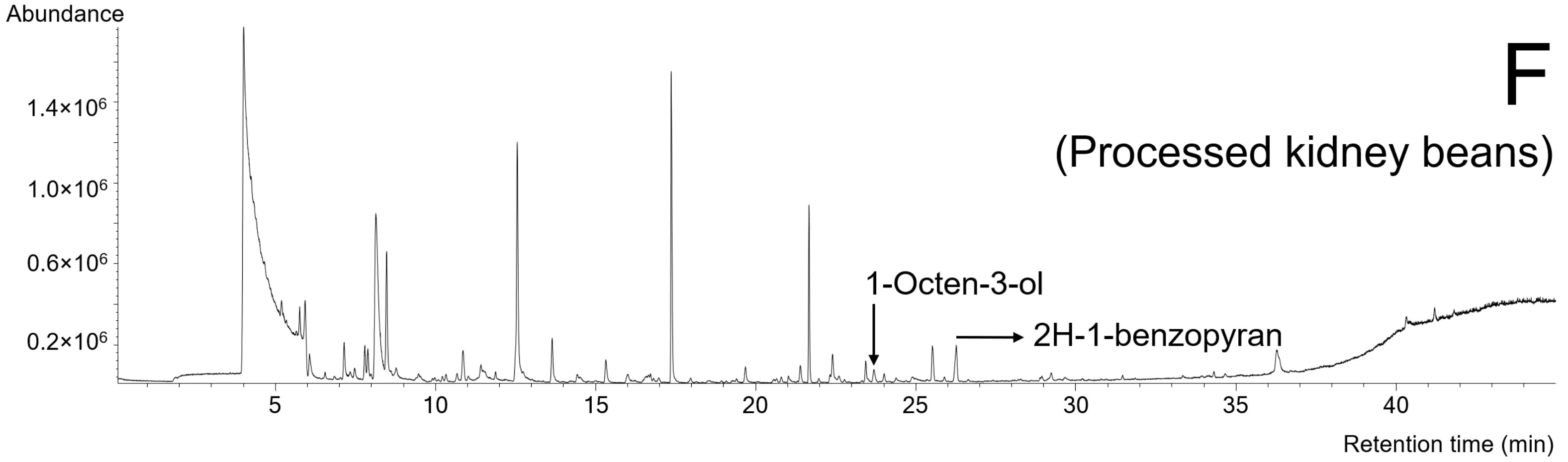

Figure S3. *Representative total ion chromatograms of cowpeas, chickpeas and kidney beans in the closed system approach. A=soaked cowpeas; B=soaked chickpeas; C=soaked kidney beans; D=processed cowpeas; E=processed chickpeas; F=processed kidney beans.*
